# Rectal Bacteria and Antibiotic Resistance in Zoo Animals in Algeria

**DOI:** 10.3390/antibiotics15070653

**Published:** 2026-06-30

**Authors:** Khayreddine Choual, Sofiane Tamendjari, Maria Francesca Peruzy, Farida Bouzebda Afri, Zoubir Bouzebda, Ridah Hadj Aissa, Nicoletta Murru, Alexis Ribas Salvador

**Affiliations:** 1 Department of Veterinary Sciences, Institute of Agricultural and Veterinary Sciences, Mohamed Cherif Messaadia University, Souk Ahras 41000, Algeria; khayreddine.choual@univ-soukahras.dz (K.C.); bfafri@yahoo.fr (F.B.A.); z.bouzebda@univ-soukahras.dz (Z.B.); 2 Laboratory of Animal Productions, Biotechnologies and Health (PABIOS), Institute of Agricultural and Veterinary Sciences, Mohamed Cherif Messaadia University, Souk Ahras 41000, Algeria; 3 Department of Veterinary Medicine and Animal Production, University of Naples Federico II, Via Federico Delpino 1, 80137 Naples, Italy; mariafrancesca.peruzy@unina.it (M.F.P.); murru@unina.it (N.M.); 4 Veterinary Service, Zoo Mansourah Park, Mansourah, Route Nationale N7, Tlemcen 13000, Algeria; hadjomarred@gmail.com; 5 Task Force on Microbiome Studies, University of Naples Federico II, 80137 Naples, Italy; 6 Department of Biology, Healthcare and Environment, Faculty of Pharmacy and Food Science, Institut de Recerca de la Biodiversitat (IRBio), University of Barcelona, 08028 Barcelona, Spain; aribas@ub.edu

**Keywords:** rectal bacterial diversity, antibiotic resistance, domestic animals, captive wild animals, Algeria

## Abstract

Background/Objectives: Healthy animals can harbor complex and diverse bacterial communities, including pathogenic taxa capable of causing disease and mortality, and individuals that are kept in zoos may act as asymptomatic carriers of a broad range of pathogens. Therefore, this study aimed to investigate the rectal bacterial microbiota of multiple animal classes (reptiles, birds, and mammals), maintained in enclosures, identify known or emerging bacterial pathogens, and characterize the antibiotic resistance profiles of the isolated strains. Methods: A total of 40 samples were collected by rectal swabbing for bacteriological analysis from 31 different animal species living in enclosures in four Algerian zoos. The isolated and identified bacterial strains were tested against certain antibiotics used in human and veterinary medicine. Statistical analyses were performed to assess differences in bacterial isolation among animal classes, sex, age categories, and zoological facilities, as well as the degree of similarity between isolated strains based on their antibiotic resistance profiles. Results: A total of 94 bacterial isolates were recovered from 40 fecal samples. Overall, the bacterial isolates belonged to seven families, 13 genera, and 16 taxa. The families identified were *Enterobacteriaceae* (50/94; 53.19%), *Staphylococcaceae* (28/94; 29.78%), *Pseudomonadaceae* (6/94; 6.38%), *Brucellaceae* (2/94; 2.12%), *Morganellaceae* (4/94; 4.25%), *Yersiniaceae* (3/94; 3.19%), and *Erwiniaceae* (1/94; 1.06%). Reptiles accounted for the highest number of isolates (37/94; 39.36%), followed by 31/94 isolates (32.97%) for birds and 26/94 isolates (27.65%) for mammals. The highest resistance rates were observed for ampicillin (AMP; 95.73%), followed by amoxicillin/clavulanic acid (AMC; 75.54%) and cephalothin (CEP; 44.90%). Lower resistance rates were detected for trimethoprim–sulfamethoxazole (SXT; 18.87%), cefotaxime (CTX; 17.41%), ceftazidime (CX; 13.67%), ciprofloxacin (CIP; 6.38%), and gentamicin (GEN; 1.05%). Conclusion: This study shows the first report that vertebrates in Algerian zoos can harbor a diverse range of cultivable bacteria, often with polymicrobial carriage. Antimicrobial resistance patterns were generally consistent with commonly used veterinary drugs. Overall, these findings contribute to a better understanding of the composition of rectal bacterial carriage in zoos.

## 1. Introduction

Bacterial diseases are a growing global concern, largely due to the alarming rise in antibiotic resistance [1]. It is generally well established that healthy animals can carry bacteria, some of which can cause serious illness and even death [2].

The spread of these bacteria in the environment increases the risk of infectious diseases emerging within animal populations [3]. These populations can serve as reservoirs for antibiotic-resistant bacteria, thereby facilitating their spread among wild and domestic animals, and potentially to humans [4], particularly in settings where species coexist closely [5]. It is increasingly recognized that the health of vertebrates is intrinsically linked to the composition of their gut microbiota [6]. This microbiota plays an essential role in regulating the immune system, energy acquisition, vitamin synthesis, and the prevention of various diseases. Nevertheless, the presence of pathogenic microorganisms, which can be transmitted via the fecal–oral route, raises concerns.

Emerging diseases, both human and animal, are often of animal origin, with approximately 70% of zoonoses worldwide originating from wild animals [7]. Recent studies have shown that wild animals can carry many pathogens without showing any clinical signs [7]. Interaction with wildlife can increase the prevalence and incidence of these pathogens [8]. Consequently, wild animals can act not only as reservoirs of virulence genes and antibiotic resistance, but also as potential sources of new genetic combinations that may pose a greater threat to humans [9].

The emergence of new pathogens in the environment often poses significant challenges for treatment [10,11]. The lack of disease surveillance in wildlife can have lasting repercussions on animal populations, leading to adverse consequences for domestic and human populations [3]. Wildlife reserves, as ecosystems less disturbed by human activity, can provide valuable indicators of contamination by antimicrobial resistance genes [12,13].

This may help mitigate the devastating effects of emerging infectious diseases, particularly in developing countries where resources for outbreak prevention and management are often limited [3].

Although the number of studies on the gut microbiota of wildlife is increasing, more than 90% of them focus on mammals [14]. Furthermore, it has been observed that grazing disturbance by wild animals significantly increases the emergence of antimicrobial resistance genes in their gut microbiota [13]. It is also worth noting that the rapid, large-scale exchange of antimicrobial resistance genes between humans, livestock, wildlife, and their food sources is a major concern [12].

The aims of the present work were to conduct the first survey of rectal bacteria in vertebrate animals (reptiles, birds, and mammals), maintained in four Algerian zoos. Specifically, this work sought to evaluate animal health status, identify known or emerging bacterial pathogens, and characterize the antibiotic resistance profiles of the isolated strains, thereby addressing a critical gap in zoo animals, especially in the African continent. 

## 2. Results

### 2.1. Prevalence of Bacterial Strains and Bacterial Species

The distribution of the isolated bacterial species according to the different animal classes is shown in Table 1. A total of 94 bacterial isolates were obtained from 40 fecal swab samples analyzed.

The sample included 13 mammals, 12 reptiles, and 15 birds. The mammal group consisted of nine males and four females, comprising eight adults and five juveniles. The reptiles included seven males and five females and were primarily adults (n = 11), with only one juvenile included. Among the birds, eleven males and four females were sampled, with a marked predominance of adults (n = 14).

Bacteriological analysis identified 94 isolates belonging to seven families, 13 genera, and 16 taxa. *Enterobacteriaceae* was the dominant family, accounting for more than half of the isolates (50/94; 53.19%), followed by the *Staphylococcaceae* (28/94; 29.78%). The other families were significantly less represented and included the *Pseudomonadaceae* (6/94; 6.38%), *Morganellaceae* (4/94; 4.25%), *Yersiniaceae* (3/94; 3.19%), *Brucellaceae* (2/94; 2.12%), and *Erwiniaceae* (1/94; 1.06%).

Reptiles were distinguished by the highest contribution to the total bacterial population, with 39.36% (37/94) of the isolates identified, as well as by the greatest taxonomic diversity. In fact, this class included six bacterial families, 11 genera, and 12 distinct species. The most frequently isolated genera were *Staphylococcus* spp. (10/12; 83.33%) and *Escherichia* (9/12; 75%), followed by *Pseudomonas* (5/12; 41.66%), *Salmonella* (4/12; 33.33%), and *Ochrobactrum* (2/12; 16.66%). The genera *Citrobacter*, *Klebsiella*, *Proteus*, *Rahnella*, *Raoultella*, and *Serratia* were detected only occasionally, each accounting for 8.33% (1/12) of the isolates.

Birds accounted for 32.97% (31/94) of all bacterial isolates and exhibited lower diversity, with five families, seven genera, and eight species identified. The genus *Escherichia* largely dominated the bacterial population (14/15; 93.33%), while *Staphylococcus* spp. (7/15; 46.66%), *Citrobacter* (4/15; 26.66%), and *Proteus* (2/15; 13.33%) were detected at lower frequencies. The genera *Pantoea*, *Pseudomonas*, and *Raoultella* were isolated sporadically, each from a single sampled individual (1/15; 6.66%).

Mammals accounted for 27.65% (26/94) of bacterial isolates and exhibited the lowest taxonomic diversity, with four families, six genera, and six species identified. The bacterial population of this class was largely dominated by *Escherichia coli* and *Staphylococcus* spp., both of which were detected in 84.61% (11/13) of the animals examined. The genera *Klebsiella*, *Kluyvera*, *Proteus*, and *Serratia* were observed sporadically, with each isolated from only a single animal (1/13; 7.69%).

Nevertheless, statistical tests revealed no significant differences in the distribution of bacteria among the various animal classes, suggesting an overall uniform distribution.

### 2.2. Antibiogram Results

#### Antibiotic Resistance Profile of Isolated Strains

The antibiotic resistance of the isolated strains is shown in Table 2. Antimicrobial susceptibility testing was performed on all 94 bacterial isolates against eight antibiotics. Overall, the highest resistance rates were observed for ampicillin (AMP), with 90/94 isolates (95.73%) resistant, followed by amoxicillin/clavulanic acid (AMC; 75.54%) and cephalothin (CEP; 44.90%). Lower resistance rates were detected for cefotaxime (CTX; 17.41%), ceftazidime (CX; 13.67%), ciprofloxacin (CIP; 6.38%), gentamicin (GEN; 1.05%), and trimethoprim–sulfamethoxazole (SXT; 18.87%).

*Escherichia coli* isolates (n = 34) showed complete resistance to AMP and AMC (100% each), moderate resistance to CEP (58.82%), and low resistance to CTX (2.94%) and SXT (14.70%), while no resistance was observed to CX, CIP, or GEN.

*Staphylococcus* spp. isolates (n = 28) exhibited universal resistance to AMP (100%), limited resistance to AMC (28.57%), and complete susceptibility to all other tested antibiotics.

*Pseudomonas aeruginosa* (n = 6) displayed a multidrug-resistant profile, with 100% resistance to AMP, AMC, CEP, CX, CTX, CIP, and SXT, while remaining fully susceptible to GEN.

*Citrobacter freundii* (n = 5) showed complete resistance to AMP and AMC, high resistance to CEP (80%), and variable resistance to CTX (60%) and CIP (20%), whereas all isolates were susceptible to CX and GEN and largely susceptible to SXT (80%).

*Salmonella enterica* subsp. *arizonae* (n = 3) exhibited a heterogeneous resistance pattern, including resistance to CTX and CIP (100%), moderate resistance to AMC (66.66%) and CEP (33.33%), and complete susceptibility to CX, GEN, and SXT.

Other *Enterobacterales*, including *Proteus mirabilis*, *Serratia liquefaciens*, *Klebsiella oxytoca*, *Salmonella* spp., *Raoultella* spp., and related genera, generally showed high resistance to β-lactam antibiotics, particularly AMP and AMC, with variable resistance to cephalosporins and marked susceptibility to aminoglycosides (GEN) and, in most cases, fluoroquinolones (CIP).

Finally, some less frequently isolated microorganisms, such as *Ochrobactrum anthropi*, were fully susceptible to all tested antibiotics, while single isolates from other genera displayed heterogeneous resistance profiles.

## 3. Discussion

The number of bacteria isolated from reptiles was higher than the number isolated from birds and mammals. In addition, certain bacterial species were found in reptiles but not in mammals and birds, other bacterial species were found in birds but not in mammals and reptiles, and other bacterial species were found in mammals but not in reptiles and birds, but there was no statistically significant difference (see the *p*-values in Table 1); the distribution of each of the 17 bacterial species was not differing significantly according to the class of animals considered, birds, reptiles or mammals. Furthermore, the chi-square independence test showed that the presence of a bacterial species did not depend on the host animal class despite the environmental, dietary, anatomical, and physiological differences among the various animal classes.

We isolated 94 bacterial strains belonging to 17 different bacterial species, namely, *Pseudomonas aeruginosa*, *Staphylococcus* spp., *Escherichia coli*, *Proteus mirabilis*, *Citrobacter freundii*, *Salmonella enterica* subsp. *arizonae*, *Serratia liquefaciens*, *Proteus vulgaris* group, *Ochrobactrum anthropi*, *Kleibsiella oxytoca*, *Rahnella aquatilis*, *Salmonella* spp., *Kluyvera* spp., *Escherichia fergusonii*, *Raoultella ornithinolytica*, *Pantoea* sp., and *Raoultella terrigena*. Many of these are potentially pathogenic and have been reported to cause infection in primates and infect humans [15].

Hirst et Halsey (2023) [3] isolated 43 bacterial strains from wild animals such as mammals, birds, and reptiles, and Kim et al. (2023) [16] reported that at least one pathogenic bacterium was present in 67.8% of the wild animals examined, and wild birds carried more pathogenic bacteria than mammals, aligning with Musa et al. (2023) [17], who reported that wild birds carried more pathogenic bacteria than wild mammals, with 72.7% and 63.6% carrying at least one pathogenic bacterium, respectively. However, research on mammals is much more extensive than on birds and reptiles [18], so wild animals may be carriers of pathogens as asymptomatic carriers [16].

The total percentage of resistance to the antibiotics tested was 95.73% for ampicillin, 75.54% for amoxicillin/clavulanic acid, 44.90% for cephalothin, 17.41% for cefoxitin, 13.67% for cefotaxime, 6.38% for ciprofloxacin, 1.05% for gentamicin, and 18.87% for trimethoprim combined with sulfamethoxazole, with only two strains of *Ochrobactrum an-thropi* sensitive to all antibiotics. These varying levels of resistance may be due to certain practices at zoos, such as the exchange of animals between zoos, the treatment of injured animals of unknown origin that are then introduced to the zoo or the purchase of animals living in an environment that may be contaminated with antibiotics; moreover, no information is known about the use of antibiotics.

*E. coli* is isolated in 85% of zoo animals with sensitivity to cefotaxime, ciprofloxacin, and gentamicin, and resistance ranging from 2.94% for cefoxitin to 100% for ampicillin and amoxicillin/clavulanic acid. Although the use of certain antibiotics in animals is prohibited in Algeria, such as amoxicillin/clavulanic acid, cefotaxime and gentamicin, resistance has been noted, and therefore the possibility of environmental contamination cannot be ruled out [17].

The presence of resistant *E. coli* species in more than two-thirds of swabs suggests that they commonly inhabit the intestinal tract of many vertebrates and can be easily transferred through close contact.

Giacopello et al. (2016) [19] and Russo et al. (2022) [20] reported that all their *E. coli* isolates were sensitive to imipenem, but other authors reported 100% resistance to first-, second-, and third-generation cephalosporins with multiple resistance [17], and 12% of wild birds were carriers of multidrug-resistant *E. coli* [21]. In Botswana, 41.3% of *E. coli* from wild animals were resistant to at least one antibiotic and 13.3% were multidrug-resistant [22], and in Saudi Arabia and Egypt, 14.4% and 39.8% of *E. coli* isolated from wild birds were multidrug-resistant, respectively [23,24], while in Brazil, 8.9% of *E. coli* isolated from peri-urban wild animals were resistant to ceftriaxone and produced ESBL [25], and in North America, 48% of *E. coli* isolated from seagulls were resistant to at least one antibiotic [26].

We isolated a total of five strains of *Salmonella* (12.5%), three of which are *Salmonella enterica*, originating from a single animal class, namely, reptiles, and exhibiting resistance to five antibiotics ranging from 33.33% for cephalothin to 100% for cefoxitin, cefotaxime, ampicillin, and amoxicillin/clavulanic acid. Low prevalences have been reported in wild birds and wild boars in Denmark (0.59%) [8], in a rehabilitation center in Switzerland (1.08%) [27], and in various wild animals in Italy (2.87%) [28], but in Brazil, Casagrande et al. (2011) [29] reported that 17% (18/106) and 17.5% of individuals of *Didelphis aurita* and *Didelphis albiventris*, respectively, are carriers of *Salmonella*. Furthermore, most of these wild animals lived in a forest surrounding a human population.

The presence of *Salmonella* has been reported in several wild animal species with varying sensitivities and resistances, ranging from sensitivity to all antibiotics to resistance to almost antibiotics tested, depending on the study, region, number of samples, and animal species in question [30,31,32,33,34]. As such, wild animals play an important role in the epidemiology of *Salmonella* spp., especially since they behave as latent asymptomatic carriers of different serotypes [35] and survive in aquatic environments for long periods of time [36].

We isolated 28 strains of *Staphylococcus* spp. from 40 wild animals, representing 70% of the total, with 100% resistance to ampicillin and 28.57% resistance to amoxicillin/clavulanic acid. Our prevalence results are higher than those reported by Aftabuddin et al. (2025) [37] (26.5%) for rodents and shrews, with resistance to oxacillin (100%) and cefixime (100%). Of the *Staphylococcus* genus, *S. aureus* is the most pathogenic [38], with an estimated cumulative prevalence of 18.5% in African, European, Asian, and American wildlife, including several animal species between 2011 and 2021 [39].

All isolated strains of *Pseudomonas aeruginosa* are resistant to all antibiotics tested (100%) except gentamicin (0%). It should be noted that five (83.33%) strains were isolated from reptiles. *Pseudomonas aeruginosa* presents various mechanisms of antimicrobial resistance, such as reduced external permeability, overexpression of efflux pumps, and production of carbapenemases [40,41], and this bacterium frequently develops resistance phenotypes to various antimicrobials due to the dissemination of plasmids and integrons [42]. *Pseudomonas aeruginosa* has the ability to persist on fomites, express potent virulence factors, and resist multiple antimicrobial agents, which has earned it the status of a healthcare-associated pathogen, and in May 2024, the WHO classified carbapenem-resistant *Pseudomonas aeruginosa* among the most dangerous bacteria requiring new control measures and the development of new antimicrobial agents [43,44].

Wild animals can act as reservoirs for zoonotic agents, from which they can emerge [45], with considerable global socioeconomic impacts [46,47], and it is important to be able to predict when, where, and in which species pathogens are most plausible to emerge. The intensive use of antimicrobials in livestock farming has led to an increase in antimicrobial resistance genes in livestock effluents and has polluted the surrounding environment [48], and consequently the plant microbiome, which represents a major route through which wild and domestic animals can be exposed to microorganisms that have acquired antimicrobial resistance genes [49,50].

Migratory birds can also disseminate resistant microorganisms across different geographical areas due to their ability to travel long distances in a short time [51], as well as other important vectors of pathogens for wild and domestic animals such as ticks [52], especially in Africa. In addition, local populations, visitors, and researchers may be responsible for some contamination of wildlife [53]. Therefore, even though wild animals are not generally treated with antimicrobials, they can acquire resistant microorganisms from various ecological niches that may harbor several antimicrobial-resistant bacteria [17].

Emerging and reemerging infectious diseases have been reported from several sources, including certain animals as reservoirs, and the evolution of microorganisms due to antimicrobial resistance poses a serious threat to global health security [7,54,55], hence the need for a “One Health” approach involving the human–animal–environment interface without neglecting wildlife.

## 4. Materials and Methods

### 4.1. Study Area and Period

The present study was conducted between November 2019 and March 2022 at four zoological facilities in Algeria: the Sétif Zoo (Wilaya de Sétif), the Parc zoologique et d’attractions “Noor & Nassim” (Wilaya de Bordj Bou Arreridj), the Zoo “Arche de Noé” (Wilaya de Ghardaia), and the Parc des reptiles (Wilaya de Ghardaia).

At the Parc des reptiles (Wilaya de Ghardaia), sampled animals belonged mainly to the class *Reptilia*, with representatives of the families *Colubridae*, *Viperidae*, *Elapidae*, *Agamidae*, *Varanidae*, *Geoemydidae*, and *Alligatoridae*, as well as one mammalian representative (Canidae) (see details in Table 3).

The Zoo “Arche de Noé” (Wilaya de Ghardaia) housed a mixed collection of mammalian and avian species, including mammals from the families *Felidae*, *Canidae*, *Equidae*, and *Leporidae*, and birds from the families *Accipitridae*, *Anatidae*, *Columbidae*, and *Phasianidae* (see details in Table 3).

At the Zoo de Sétif (Wilaya de Sétif), sampled animals included representatives of all three classes (*Mammalia*, *Aves*, and *Reptilia*), with families *Accipitridae*, *Corvidae*, and *Struthionidae* among birds; *Camelidae*, *Caviidae*, *Cercopithecidae*, and *Equidae* among mammals; and *Alligatoridae* and *Colubridae* among reptiles (see details in Table 3).

The Parc zoologique et d’attractions “Noor & Nassim” (Wilaya de Bordj Bou Arreridj) hosted predominantly avian species, including the families *Accipitridae*, *Phasianidae*, *Columbidae*, *Corvidae*, and *Strigidae*, as well as mammalian representatives from the families *Bovidae* and *Equidae*, and one reptile from the family *Agamidae* (see details in Table 3).

### 4.2. Sample Collection

The sample size was estimated using the standard formula employed in descriptive epidemiological studies examining a proportion or prevalence.

Formula used:

n = [Z^2^ × *p* × (1 − *p*)] ÷ d^2^;

where n represents the minimum sample size; Z: value corresponding to a 95% confidence interval; *p*: expected prevalence (*p* = 50%); d: precision (margin of error: 15%).

In the absence of prevalence data from previous studies, the expected prevalence in the population is set at 0.5 to maximize n (sample size) [56].

n = [Z^2^ × *p* × (1 − *p*)] ÷ d^2^, i.e., n = [(1.96)^2^ × 0.5 × (1 − 0.5)] ÷ (0.15)^2^ = 42.7 (≈43 animals).

In our study, the total population (finite population) was approximately 700 animals (the exact number is difficult to determine because there are exchanges, purchases, sales, and mortalities); thus, the sample size was calculated using the formula for a finite population with an adjusted n.

adjusted n = n/(1 + [(n − 1)/N)] = 42.7/(1 + [(42.7 − 1)/700] = 40.3 ≈ 40 animals.

For the sampling, a stratified random sampling method with equal allocation was used to ensure that every animal has an equal chance of being selected.

A total of 40 samples (10 by zoo) were collected by rectal swabbing from three different animal classes, namely, reptiles (n = 12), mammals (n = 13), and birds (n = 15), belonging to 22 animal families, 29 animal genera, and 31 different animal species (Table 3).

Animals underwent a comprehensive clinical examination, including general physical assessment, measurement of rectal body temperature, and cardiac and respiratory auscultation before sampling in order to assess their health status.

### 4.3. Sample Processing

Swabs were transported at 4 °C to the ESSALEM laboratory in Ghardaïa and processed within 24 h after sampling.

Nine milliliters of sterile physiological water were added to the sterile swab (Swab Sticker Female, CELABO CE). The swab was closed and placed on the vortex for one (01) minute, the swab was pressed against the wall of the tube before being removed to be seeded directly onto six (06) different culture media (BIOKAR, Allonne, France): Nutrient Agar, MacConkey Agar, Columbia Agar with blood, Hektöen Enteric Agar, Salmonella–Shigella Agar, and Mannitol Salt Agar. Next, three decimal dilutions were prepared from the stock solution obtained, and one milliliter of each solution is seeded in the different culture media to be incubated at 37 °C for 24, 48, and 72 h in order to obtain several different colonies for macroscopic identification, isolation, Gram staining, and biochemical identification using an API 20E strip (BioMérieux, Marcy-l'Étoile, France) and/or reagents.

Biochemical tests were performed using the API 20E strip following the manufacturer’s instructions.

All identified strains were seeded, subcultured and re-seeded four to five times in their selective media in order to obtain purified strains. The purified strains were stored under refrigeration temperature [57] and transported in a cooler at 4 °C to the Animal Production, Biotechnology and Health Laboratory (PABIOS) at the Institute of Agronomic and Veterinary Sciences at Mohamed Cherif Messaidia University, Souk Ahras, Algeria, for antibiogram testing.

### 4.4. Evaluation of Antibiotic Resistance

The antimicrobial susceptibility of the isolates was determined by the disk-diffusion method on Mueller–Hinton Agar (BIOKAR, Allonne, France) following the Clinical and Laboratory Standards Institute (CLSI) recommendations [58].

The following antibiotics (Liofilchem, Roeseto, Italy) were used: ampicillin (AMP) (10 μg); amoxicillin/clavulanic acid (AMC) (20 μg/10 μg); cephalothin (CEP) (30 μg); cefoxitin (CX) (30 μg); cefotaxime (CTX) (30 μg); ciprofloxacin (CIP) (5 μg); gentamicin (GEN) (30 μg); trimethoprim + sulfamethoxazole (SXT) (1.25 μg/23.75 μg).

### 4.5. Statistical Tests

The statistical analyses of the results obtained were performed using STATISTICA 7 software (Statsoft, Maisons-Alfort, France), namely, the chi-square test (χ^2^) and one-way analysis of variance.

The chi-square test was used to study the distribution of bacterial species across different animal classes. To limit the inflation of the Type I error rate associated with multiple comparisons, a Bonferroni correction was applied.

The relationship between the type of antibiotic and the degree of antibiotic resistance was analyzed using one-way ANOVA.

Statistical significance was set at *p* < 0.05.

## 5. Conclusions

In conclusion, this study presents first data on animals from four Algerian zoos, showing a diverse range of cultivable bacteria, often with polymicrobial carriage. Differences observed among mammals, birds, and reptiles suggest that host characteristics and environmental exposure may influence bacterial composition. The detection of *Salmonella*, particularly in reptiles, supports previous evidence that these animals can act as reservoirs of potentially zoonotic bacteria. Antimicrobial resistance patterns were generally consistent with commonly used veterinary drugs, while most critically important antimicrobials remained effective. Overall, these findings provide baseline information that may contribute to a better understanding of bacterial carriage in wildlife.

## Figures and Tables

**Table 1:** Prevalence of bacterial strains isolated by animal class.

Bacteria	Number of Bacteria Isolated n = 40 (94 Bacteria)	Mammalian n= 13 (26 Bacteria)	Reptile n = 12 (37 Bacteria)	Bird n = 15 (31 Bacteria)	*p*-Value
*Pseudomonas aeruginosa*	06 (15%)	0 (0%)	5 (41.66%)	1 (6.66%)	0.06594
*Staphylococcus* spp.	28 (70%)	11 (84.61%)	10 (83.33%)	7 (46.66%)	0.2401
*Escherichia coli*	34 (85%)	11 (84.61%)	9 (75%)	14 (93.33%)	0.1527
*Proteus mirabilis*	3 (7%)	1 (7.69%)	0 (0%)	2 (13.33%)	0.3251
*Citrobacter freundii*	5 (12.5%)	0 (0%)	1 (8.33%)	4 (26.66%)	0.07093
*Salmonella enterica* subsp. *arizonae (Salmonella choleraesuis* subsp. *arizonae)*	3 (7.5%)	0 (0%)	3 (25%)	0 (00%)	0.08821
*Serratia liquefaciens*	2 (5%)	1 (7.69%)	1 (8.33%)	0 (0%)	0.2016
*Proteus vulgaris*	1 (2%)	0 (0%)	1 (8.33%)	0 (0%)	0.4529
*Ochrobactrum anthropi*	2 (5%)	0 (0%)	2 (16.66%)	0 (0%)	0.2016
*Klebsiella oxytoca*	2 (5%)	1 (7.69%)	1 (8.33%)	0 (0%)	0.5671
*Rahnella aquatilis*	1 (2.5%)	0 (0%)	1 (8.33%)	0 (0%)	0.4529
*Salmonella* spp.	2 (5%)	0 (0%)	2 (16.66%)	0 (0%)	0.2016
*Kluyvera* spp.	1 (2.5%)	1 (7.69%)	0 (0%)	0 (0%)	0.2616
*Escherichia fergusonii*	1 (2.5%)	0 (0%)	0 (0%)	1 (6.66%)	0.3698
*Raoultella ornithinolytica*	1 (2.5%)	0 (0%)	0 (0%)	1 (6.66%)	0.3698
*Pantoea* sp.	1 (2.5%)	0 (0%)	0 (0%)	1 (6.66%)	0.3698
*Raoultella terrigena (* *Klebsiella terrigena* *)*	1 (2.5%)	0 (0%)	1 (8.33%)	0 (0%)	0.4529
*p*-value = 0.0889					

**Table 2:** Antibiotic resistance of isolated strains against ampicillin (AMP), amoxicillin/clavulanic acid (AMC), cephalothin (CEP), cefoxitin (CX), cefotaxime (CTX), ciprofloxacin (CIP), gentamicin (GEN), and trimethoprim + sulfamethoxazole (SXT).

Identified species	NS	AMP ^g^	AMC ^f^	CEP ^e^	CX ^a^	CTX ^d^	CIP ^c^	GEN ^b^	SXT ^a^
*Pseudomonas aeruginosa*	6	100% ^a^	100% ^a^	100% ^a^	100% ^a^	100% ^a^	100% ^a^	0% ^b^	100% ^a^
*Staphylococcus* spp.	28	100% ^a^	28.57% ^b^	0% ^c^	0% ^c^	0% ^c^	0% ^c^	0% ^c^	0% ^c^
*Escherichia coli*	34	100% ^a^	100% ^a^	58.82% ^b^	2.94% ^c^	0% ^c^	0% ^c^	0% ^c^	14.70% ^d^
*Proteus mirabilis*	3	100% ^a^	100% ^a^	66.66% ^b^	0% ^c^	0% ^c^	0% ^c^	33.33% ^d^	33.33% ^c^
*Citrobacter freundii*	5	100% ^a^	100% ^a^	80% ^b^	60% ^c^	20% ^d^	0% ^e^	0% ^e^	20% ^d^
*Salmonella enterica* subsp. *arizonae*	3	33.33% ^a^	66.66% ^b^	33.33% ^a^	100% ^c^	100% ^c^	0% ^d^	0% ^d^	0% ^d^
*Serratia liquefaciens*	2	100% ^a^	100% ^a^	50% ^b^	0% ^c^	0% ^c^	0% ^c^	0% ^c^	0% ^c^
*Proteus vulgaris* group	1	100%	100%	0%	0%	0%	0%	0%	0%
*Ochrobactrum anthropi*	2	0%	0%	0%	0%	0%	0%	0%	0%
*Kleibsiella oxytoca*	2	100% ^a^	100% ^a^	50% ^b^	0% ^c^	0% ^c^	0% ^c^	0% ^c^	50% ^c^
*Rahnella aquatilis*	1	100%	100%	0%	0%	0%	0%	0%	0%
*Salmonella* spp.	2	100% ^a^	100% ^a^	100% ^a^	50% ^b^	50% ^b^	0% ^c^	0% ^c^	0% ^c^
*Kluyvera* spp.	1	100%	100%	100%	0%	100%	0%	0%	100%
*Escherichia fergusonii*	1	100%	100%	100%	0%	0%	0%	0%	0%
*Raoultella ornithinolytica*	1	100%	100%	100%	100%	0%	0%	0%	0%
*Pantoea* sp.	1	100%	100%	100%	0%	0%	0%	0%	100%
*Raoultella terrigena*	1	100%	100%	100%	100%	100%	0%	0%	100%
Total resistance	94	95.73%	75.54%	44.90%	17.41%	13.67%	6.38%	1.05%	18.87%

NS: Number of strains. Within each row the same superscript (^a–g^) suggests no significant pairwise difference, while the existence of a different superscript indicates a statistically significant one.

**Table 3:** Number of animals studied by animal class, animal family, animal genus, animal species and their origin.

Animal Class (03)	Family (22)	Genus (29)	Species (31)	Common Name	Number of Animals (40)	Origin of Animals
*Aves*	*Accipitridae*	*Aquila*	*Aquila chrysaetos*	Golden Eagle	1	Injured and treated
		*Circaetus*	*Circaetus cinereus*	Brown Snake Eagle	1	Injured and treated
		*Gyps*	*Gyps fulvus*	Griffon Vulture, Eurasian Griffon, Eurasian Griffon Vulture	1	Born in captivity
		*Neophron*	*Neophron percnopterus*	Egyptian Vulture, Egyptian Eagle	2	Exchange, injured and treated
	*Anatidae*	*Anas*	*Anas platyrhynchos*	Mallard, Common Mallard, Northern Mallard	1	Injured and treated
	*Columbidae*	*Columba*	*Columba livia*	Curly Pigeon, Pouter pigeon	2	Born in captivity
	*Corvidae*	*Corvus*	*Corvus corax*	Common Raven, Northern Raven, Raven	2	Injured and treated
	*Phasianidae*	*Gallus*	*Gallus gallus domesticus*	Brahma chicken	1	Born in captivity
		*Lophura*	*Lophura nycthemera*	Silver Pheasant	1	Purchase
		*Pavo*	*Pavo cristatus*	Indian Blue Peafowl, Indian Peafowl, Common Peafowl, Blue Peafowl, Peafowl	1	Born in captivity
	*Strigidae*	*Bubo*	*Bubo ascalaphus*	Pharaoh Eagle-Owl	1	Injured and treated
	*Struthionidae*	*Struthio*	*Struthio camelus*	Common Ostrich, Ostrich	1	Purchase
*Mammalia*	*Bovidae*	*Capra*	*Capra aegagrus hircus*	Damascus Goat	1	Purchase
	*Camelidae*	*Camelus*	*Camelus dromedarius*	Dromedary, Arabian Camel	1	Born in captivity
	*Canidae*	*Vulpes*	*Vulpes zerda*	Fennec Fox	2	Born in captivity
	*Caviidae*	*Cavia*	*Cavia porcellus*	Domestic Guinea Pig, Guinea Pig, Domestic Cavy	1	Born in captivity
	*Cercopithecidae*	*Macaca*	*Macaca sylvanus*	Barbary Macaque, Barbary Ape, Magot	1	Born in captivity
	*Equidae*	*Equus*	*Equus ferus caballus* or *Equus caballus*	Barb Horse (02), Miniature Horse	3	Born in captivity
	*Felidae*	*Panthera*	*Panthera tigris*	Tiger	3	Born in captivity
	*Leporidae*	*Oryctolagus*	*Oryctolagus cuniculus*	European Rabbit	1	Purchase
*Reptilia*	*Agamidae*	*Uromastyx*	*Uromastyx alfredschmidti*	Ebony Mastigure, Schmidt’s Mastigure, Schmidt’s Spiny-Tailed Lizard	1	Injured and treated
			*Uromastyx dispar* ssp. *Maliensis*	Malian Mastigure, Mali Spiny-tailed Lizard	1	Injured and treated
	*Alligatoridae*	*Alligator*	*Alligator mississippiensis*	American Alligator, Mississippi Alligator	1	Exchange
	*Colubridae*	*Hemorrhois*	*Hemorrhois algirus*	Algerian Whip Snake	1	Purchase
			*Hemorrhois hippocrepis*	Horseshoe Whip Snake	2	Purchase
		*Spalerosophis*	*Spalerosophis dolichospilus*	Mograbin Diadem Snake	1	Purchase
	*Elapidae*	*Naja*	*Naja haje*	Egyptian Cobra	1	Purchase
	*Geoemydidae*	*Mauremys*	*Mauremys leprosa*	Mediterranean Pond Turtle, Mediterranean Turtle, Mediterranean terrapin, Spanish Terrapin, Mediterranean Stripe-necked Terrapin	1	Purchase
	*Varanidae*	*Varanus*	*Varanus griseus*	Desert Monitor Lizard	1	Purchase
	*Viperidae*	*Cerastes*	*Cerastes cerastes*	Horned Viper, Desert Horned Viper, Saharan Horned Viper, Algerian Horned Viper, Horn Viper	1	Injured and treated
		*Macrovipera*	*Macrovipera lebetina*	Blunt-nosed Viper, Lebetine Viper, Levant Viper	1	Purchase

## Data Availability

Data are contained within the article.
